# Construction of 12 EST libraries and characterization of a 12,226 EST dataset for chicory (*Cichorium intybus*) root, leaves and nodules in the context of carbohydrate metabolism investigation

**DOI:** 10.1186/1471-2229-9-14

**Published:** 2009-01-28

**Authors:** Nicolas Dauchot, Dominique Mingeot, Bénédicte Purnelle, Céline Muys, Bernard Watillon, Marc Boutry, Pierre Van Cutsem

**Affiliations:** 1 Unité de Recherche en Biologie Cellulaire et Moléculaire Végétale (URBV), Facultés Universitaires Notre-Dame de la Paix Namur (FUNDP), Rue de Bruxelles 61, 5000 Namur, Belgium; 2 Département de Biotechnologie, Centre wallon de Recherches agronomiques (CRA-W), Chaussée de Charleroi 234, 5030 Gembloux, Belgium; 3 Institut des Sciences de la Vie, Université Catholique de Louvain (UCL), Croix du Sud 2/20, 1348 Louvain-La-Neuve, Belgium

## Abstract

**Background:**

The industrial chicory, *Cichorium intybus*, is a member of the *Asteraceae *family that accumulates fructan of the inulin type in its root. Inulin is a low calories sweetener, a texture agent and a health promoting ingredient due to its prebiotic properties. Average inulin chain length is a critical parameter that is genotype and temperature dependent. In the context of the study of carbohydrate metabolism and to get insight into the transcriptome of chicory root and to visualize temporal changes of gene expression during the growing season, we obtained and characterized 10 cDNA libraries from chicory roots regularly sampled in field during a growing season. A leaf and a nodule libraries were also obtained for comparison.

**Results:**

Approximately 1,000 Expressed Sequence Tags (EST) were obtained from each of twelve cDNA libraries resulting in a 12,226 EST dataset. Clustering of these ESTs returned 1,922 contigs and 4,869 singlets for a total of 6,791 putative unigenes. All ESTs were compared to public sequence databases and functionally classified. Data were specifically searched for sequences related to carbohydrate metabolism. Season wide evolution of functional classes was evaluated by comparing libraries at the level of functional categories and unigenes distribution.

**Conclusion:**

This chicory EST dataset provides a season wide outlook of the genes expressed in the root and to a minor extent in leaves and nodules. The dataset contains more than 200 sequences related to carbohydrate metabolism and 3,500 new ESTs when compared to other recently released chicory EST datasets, probably because of the season wide coverage of the root samples. We believe that these sequences will contribute to accelerate research and breeding of the industrial chicory as well as of closely related species.

## Background

The industrial chicory (*Cichorium intybus *L.) is a fructan accumulating crop member of the *asteraceae *family. Fructan is a storage carbohydrate used as an alternative to starch by approximately 15% of all flowering plants [1]. Inulin, a linear polymer made of β-2,1 linked fructosyl units with a single terminal glucose, is stored in the taproot of chicory. While short oligofructans are used as low calories sweeteners, long inulin chains are dietary fibers also used by the agro-industry as low calories fat substitute. They are considered as health promoting ingredients since they selectively stimulate the growth of beneficial colonic microflora which in turn produces inulin degradation by-products that improve blood parameters [2,3], reduce pathogenic bacteria [4], increase calcium fixation and reduce the risks of developing colon cancers [5].

The first model describing the fructan biosynthesis pathway was proposed by Edelman and Jefford (1968) from studies performed in *Helianthus tuberosus *[6], another inulin accumulating crop. Inulin biosynthesis requires the sequential action of two enzymes: the sucrose:sucrose-1 fructosyltransferase (1-SST) and the fructan:fructan-1 fructosyltransferase (1-FFT). During the initiation step, the enzyme 1-SST catalyses the transfer of a fructosyl unit from a sucrose molecule (disaccharide: Glu-Fru) to a second sucrose molecule acting as acceptor of fructosyl units to produce 1-kestose (Glu-Fru-Fru), the smallest fructan molecule and free glucose. During the elongation step, the trisaccharide 1-Kestose is further elongated through the activity of the enzyme 1-FFT using 1-kestose or fructans of higher polymerization degree (DP) as source of fructosyl units (GFF_n1 _+ GFF_n2 _→ GFF_n1-1 _+ GFF_n2+1_). Alternatively, 1-FFT can use free fructose as acceptor, producing fructosyl only oligosaccharides of the inulose type (glucose free inulin) [7]. This model of inulin biosynthesis is supported by *in vitro *[8] and transgenic studies [9,10] and is widely accepted by the scientific community despite occasional criticism [11].

In industrial chicory, the average inulin chain length and yield varies during the growing season and between cultivars [12-14]. With the modification of European sugar policy, short inulin chains are now considered as undesirable by-products and breeders look for cultivars accumulating long inulin chains. Beside environmental factors, the molecular components of inulin chain length and content variability among cultivars are still unknown even though some strong candidates are proposed. It was demonstrated that the difference of inulin chain length observed between *Helianthus tuberosus *and *Cynara scolymus *is related to the properties of their respective 1-FFT [10]. In chicory, exposure to cold temperature decreases the transcription and the activity of 1-SST, while transcription and activity of inulin degrading enzymes are increased resulting in a global shortening of inulin chains length [15]. While inulin biosynthesis and degrading enzymes are clear targets to investigate the molecular bases of inter-varietal variability of inulin properties, other genetic factors could also be involved such as genes of the photosynthetic pathways, sugar transporters, receptors, perception of cold and water availability, transcription factors, *etc*.

Despite extensive biochemical investigations of inulin metabolism during the last decades, very limited chicory nucleotide sequence resources were publicly available until very recently. This is why we decided to generate 12 cDNA libraries, mainly from root tissues sampled throughout the growing season. These new nucleotide databases from genes expressed in the root will help to investigate carbohydrate metabolism in fructan accumulating crops.

## Results

### Sequencing and clustering

**Figure 1:**
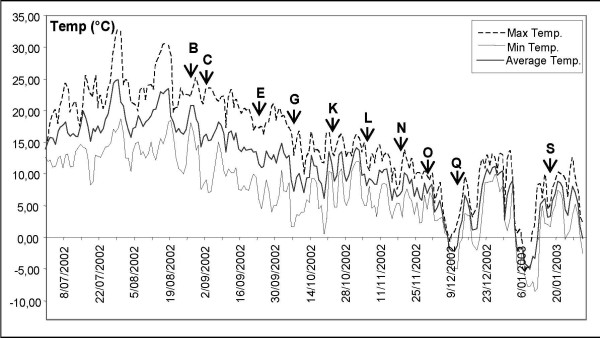
**Chicory root tissues sampling dates**. Evolution of minimal, average and maximal temperatures during growing season 2002 in Gembloux (5030-Belgium). The sampling dates used for cDNA library production are indicated with arrows and corresponding reference letters.

All cDNA libraries were non-normalized. Ten root libraries were generated from a single chicory cultivar (Arancha) harvested during growing season 2002 (figure 1), providing a season-wide overview of the genes expressed in chicory roots. The first letter (R-root, F-leaf, N-nodule) refers to the organ from which RNA were extracted while the second letter refers to the sampling date (see figure 1).

Two cDNA libraries were also produced from leaves and nodules. The nodule library was obtained from tissue culture of witloof chicory. Out of each cDNA library, approximately 1,000 clones were sequenced from their 3'end, reaching a total of 12,524 3'EST sequences. Out of 12,524 chicory ESTs, 12,226 sequences remained after removal of repeated sequences, vector and organelle sequences which still represent more than 7.10^6 ^bp. In the future, these sequences will be referred to as the "PHYTOMOL" dataset. All the EST sequences generated during this research are accessible at NCBI under accession numbers [GenBank: FL670599] to [GenBank: FL682824].

A unigene dataset was generated using the EGassembler bioinformatics pipeline [16]. This resulted in the detection of 0.1% of transposable elements, namely 13 DNA transposons and 53 retro-elements, mainly Copia (41) and Gypsy (11) LTR elements but also one LINE. Over 800 simple sequence repeats were detected, representing 0.52% of the sequences, while low complexity repeats accounted for 0.62%. In total, 1.24% of the 7,451,304 sequenced base pairs were repetitive elements.

The EGassembler online clustering pipeline returned 1,922 contigs (average size 770 bp) and 4,869 singlets (average size 595 bp), providing a total of 6,791 chicory unigenes (analyses performed with default parameters). When considering each dataset independently, the ratio of "singlets" vs "sequences included in contigs" is rather similar, ranging from 56.2% for the EST library "FC" to 74.8% for "RG", with an average of 70.4% for the twelve libraries (table 1).

**Table 1:** Frequency of singlets within the twelve chicory EST datasets

Library	Singleton	Total ESTs	% of Singlets
RB	674	967	69.7

FC	559	994	56.2

RC	784	1,020	76.8

RE	739	1,005	73.5

RG	747	999	74.8

RK	735	1013	72.5

RL	678	988	68.6

RN	698	1,003	69.6

RO	861	1,185	72.6

RQ	734	993	73.9

RS	706	1091	64.7

NO	686	968	70.9

**Average**	**717**	**1,019**	**70.4%**

For each EST dataset singlets and contigs were sorted using the EGassembler bioinformatics pipeline.

### The PHYTOMOL dataset as a source of SNPs

In order to quantify the SNP discovery rate in the PHYTOMOL database, a bio-informatic analysis was performed using the SNP/indel discovery pipeline developed by the CGPDB [16] with further manual verifications. This pipeline consists of 24 steps containing scripts compatible with third party open source software. Starting from the entire PHYTOMOL dataset, the first steps generated a set of 1,546 contigs which were later searched for SNP polymorphisms. The average size of these contigs was 781 bp as compared to 635 bp of the 7,145 singlets. The number of contigs and singlets is clearly different when compared to the clustering results returned by the EGassembler pipeline (respectively 1,922 contigs and 4,869 singlets), indicating that the clustering parameters might be more stringent in order to reduce the interference of paralogs. The number of sequences in these contigs ranged from 2 to 77 with an average of 3.46 sequences per contig. The final results are reported in table 2. Since EST sequences are subject to sequencing errors, different significance thresholds were defined. The MM column reports the number of SNPs detected in the dataset by the CGPDB pipeline: this number is clearly biased by sequencing errors. The MM.good column refers to the number of SNPs present at least twice in a contig, while the last column indicates the number of SNPs detected after manual editing of the MM.good results, only considering SNP present more than twice (restricting the analyses to 653 contigs containing at least three sequences) and located outside of the 5' and 3' ends of the contigs or of low quality regions. This manual editing of the contigs drastically reduced the number of SNPs selected down to 294 SNPs in 64 contigs, which represents an average of 4.6 SNPs per contig and an average frequency of 1 SNP per 198 bp. Visual inspection of these contigs coupled to the average SNP frequency indicates that these SNPs are more than likely "real" SNPs and not artifacts produced by clustering paralogous sequences, which reinforces the assumption concerning the stringency of the CGPDB clustering parameters.

**Table 2:** Results of the *in silico *SNP detection

	MM	MM.good	Manual selection
Number of contigs (CAP3)	1,546	1,546	653

SNP selection criterion (occurrence)	all SNPs	at least 2 times	more than 2 times

Number of SNPs containing contigs	1,417	255	64

Total number of SNP detected	43,656	1,886	294

Average SNP frequency in contigs	30.8 (1/25 bp)	7.4 (1/105 bp)	4.6 (1/198 bp)

The SNP/indel discovery pipeline generates contigs. Depending on the selection stringency (MM, MM.good and Manual selection), the number of contigs included in the analyses ranged from 1,546 down to 653 when the selection was restricted to clusters containing a least three sequences (manual). Within these contigs, those effectively containing SNPs are reported as well as the total number of SNPs detected and the average SNP frequency in the contigs.

### Functional characterization of the unigene dataset

**Figure 2:**
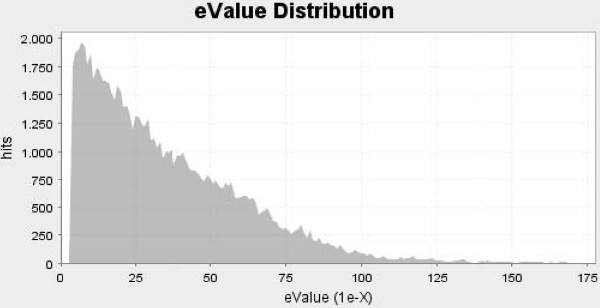
**Characterization of the unigene dataset annotation results**. eValue after BLASTX homology search performed on 6.813 chicory unigenes.

**Figure 3:**
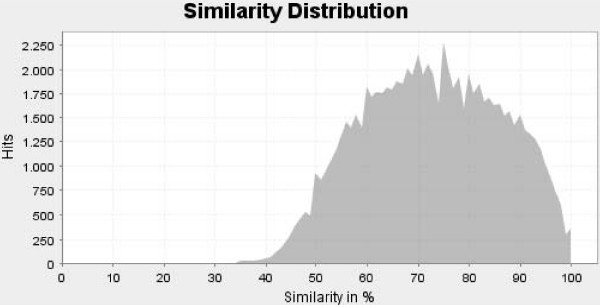
**Characterization of the unigene dataset annotation results**. Similarity distribution after BLASTX homology search performed on 6.813 chicory unigenes.

The 6,791 industrial chicory unigenes were searched for homology (BLASTX) against the NCBI nr database. These analyses were performed with the Blast2GO software [17] with a minimal eValue set to 10^-4^. Among unigenes, 4,811 sequences (70.8%) returned a significant BLAST result. The eValue for BLASTX results ranged from 1.10^-04 ^to 1.10^-176^. For 4,303 (89%) BLASTX results, the eValue was lower than 1.10–10, among which 1,678 were lower than 10–50. Figures 2 and 3 present eValue and similarity distribution charts.

Out of the 4,811 positive BLASTX results, 1,497 (31%) refer to hypothetical (569), putative (249) or unknown/unnamed (679) gene products.

To classify the sequences in functional categories, the BLAST results passed through a mapping and an annotation procedure (Blast2GO). Parameters set up for the annotation process were reviewed previously (refer to the "evaluation section" of the Blast2GO website [17]. For these analyses, the eValue was set to 1.10^-05^, Annotation Cut Off "45" and GO Weight "10". These parameters gave the best balance between annotation rate (80%) and accuracy (70% at branch level). Gene ontology functional classification terms (GO) could be retrieved for 4,608 sequences (95.8%) during the "mapping" procedure performed by Blast2GO.

Out of 4,608 mapped unigenes (BLASTX results and GO term), 3,580 (77.7%) could be functionally annotated (GO consensus and E.C. number). The initial annotation was converted into a plant specific simplified annotation (plant GoSlim).

**Figure 4:**
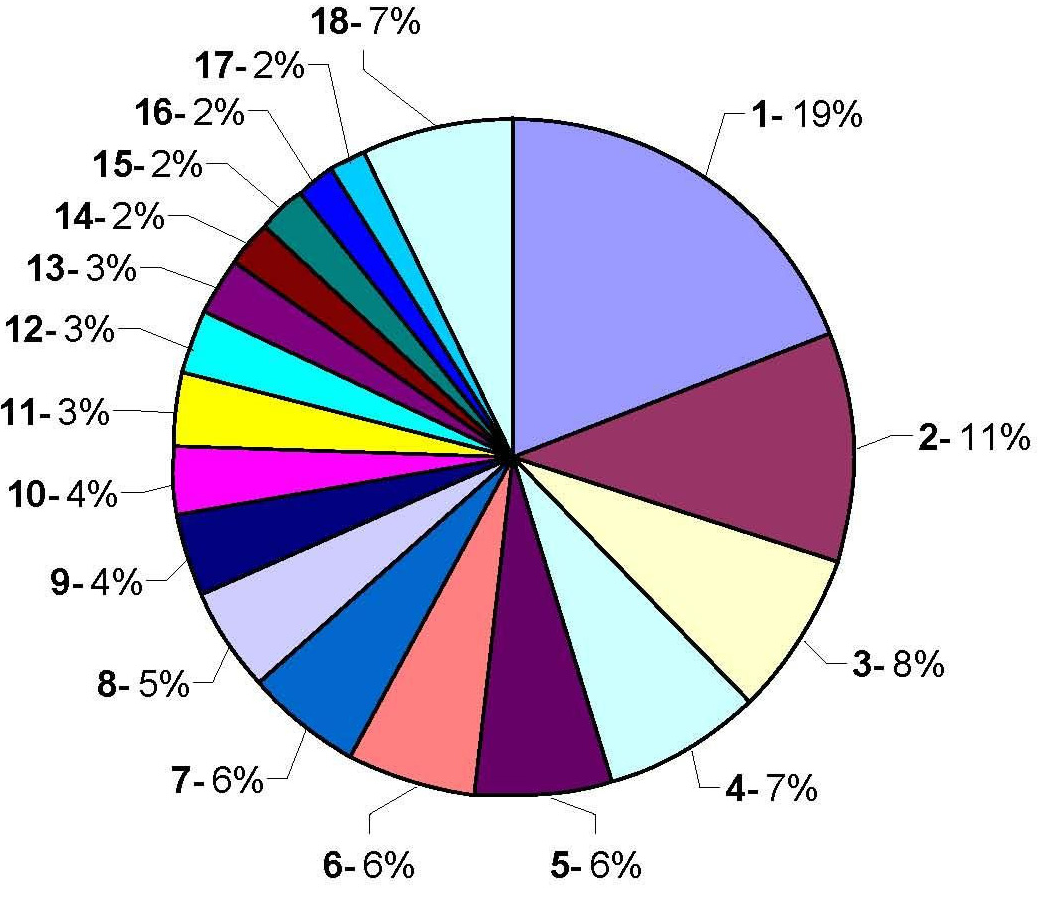
**Functional classification. Biological process**. Relative abundance of biological processes represented in the PHYTOMOL unigene dataset, according to the Gene Ontology annotation database. 1-Transport, 2-Response to abiotic stimulus, 3-Response to stress, 4-Translation, 5-Protein modification, 6-Transcription, 7-Response to endogenous stimulus, 8-Signal transduction, 9-Catabolic process, 10-Carbohydrate metabolic process, 11-Response to biotic stimulus, 12-Amino acid and derivative metabolic process, 13-Lipid metabolic process, 14-Electron transport, 15-DNA metabolic process, 16-Embryonic development, 17-Secondary metabolic process, 18-Others.

**Figure 5:**
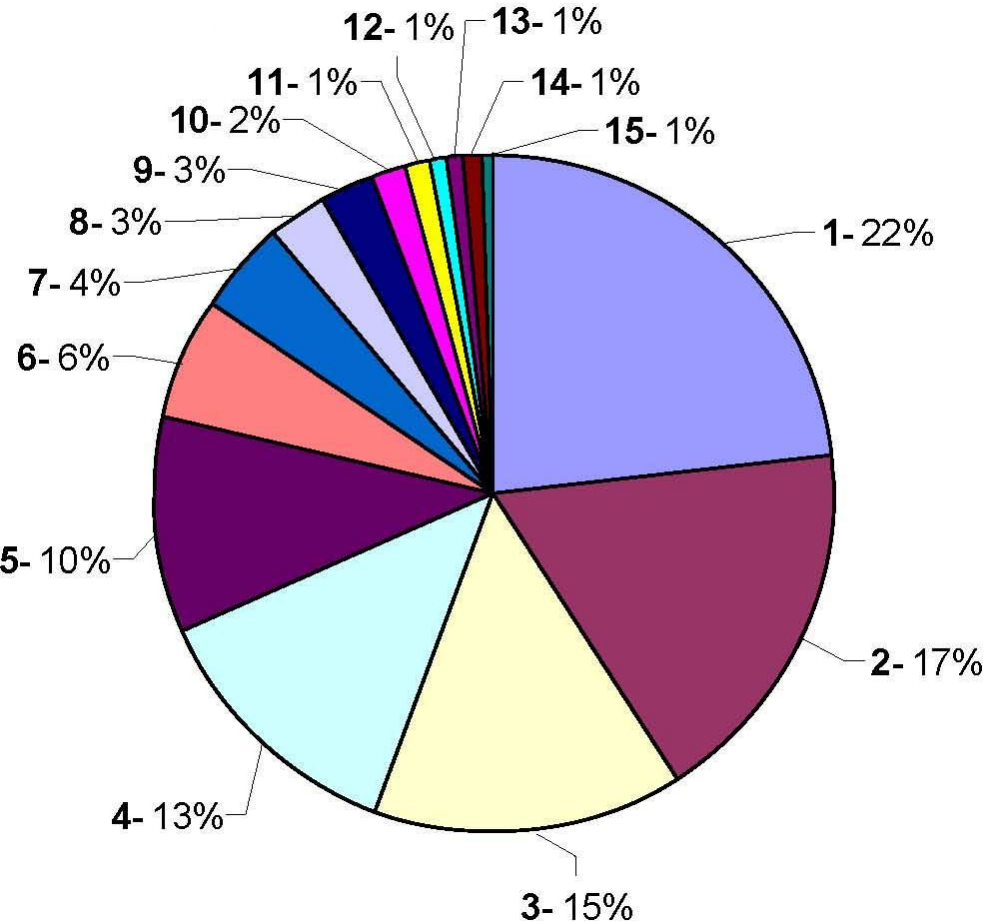
**Functional classification. Molecular function**. Relative abundance of molecular functions represented in the PHYTOMOL unigene dataset, according to the Gene Ontology annotation database. 1-Protein binding, 2-Nucleotide binding, 3-Kinase activity, 4-Transporter activity, 5-Structural molecule activity, 6-RNA binding, 7-Transcription factor activity, 8-Enzyme regulator activity, 9-Translation factor activity, 10-Lipid binding, 11-Nuclease activity, 12-Motor activity, 13-Carbohydrate binding, 14-Chromatin binding, 15-Receptor activity.

Figures 4 and 5 summarize the relative abundance of biological processes and molecular functions represented in this unigene dataset, according to the Gene Ontology annotation database [18]. Molecular function refers to activities such as catalytic or binding activities, while biological processes make reference to a series of ordered molecular functions such as signal transduction or sugar transport.

### Sequences related to carbohydrate metabolism

One of the scopes of this research was to isolate genes related to carbohydrate metabolism. When searching the functional annotation results (4,608 "mapped" unigenes), 117 were related to carbohydrate metabolic processes (biological process), while 12 matched the carbohydrate binding category of molecular functions. Out of these 129 unigenes, 90 could be positioned on Kegg (Kyoto Encyclopedia of Genes and Genomes) metabolic pathways maps. These 90 sequences refer to 40 E.C. categories (Enzyme Commission) and take part in 47 carbohydrate related metabolic pathways (Tables 3 and 4).

**Table 3:** E.C. classes detected among chicory unigenes related to carbohydrates

E.C. classes	Examples	Abundance
1.1.1.133	dTDP-4-dehydroramnose reductase	2

1.1.1.2	alcohol dehydrogenase (NADP+)	2

1.1.1.27	L-lactate dehydrogenase	1

1.1.1.49	glucose-6-phosphate dehydrogenase	2

1.2.1.12	glyceraldehyde-3-phosphate dehydrogenase	6

1.3.99.1	succinate dehydrogenase	1

1.6.1.1	NAD(P)+ transhydrogenase (B-specific)	1

2.2.1.1	transketolase	2

2.3.3.8	ATP citrate synthase	2

2.4.1.1	phosphorylase	2

2.4.1.224	4-alpha-N-acetylgucosaminyltransferase	2

2.4.1.225	4-beta-glucurnosyltransferase	2

2.6.1.1	aspartate transaminase	1

2.7.1.40	pyruvate kinase	6

2.7.1.6	galactokinase	1

2.7.2.3	phosphoglycerate kinase	1

2.7.7.13	mannose-1-phosphate guanyltransferase	1

2.7.7.7.27	ADP glucose pyrophosphorylase	1

2.7.7.9	UTP-glucose pyrophosphorylase	2

3.1.3.11	fructose-bisphosphatase	3

3.1.3.12	trehalose-phosphatase	1

3.1.3.46	fructose-2,6-bisphosphate 2-phosphatase	2

3.2.1.1	alpha-amylase	4

3.2.1.14	chitinase	1

3.2.1.15	polygalacturonase	7

3.2.1.4	cellulase	1

3.6.3.6	H+-exporting ATPase	1

4.1.1.49	phosphoenolpyruvate caroboxylase	2

4.1.2.13	fructose-bisphosphate aldolase	8

4.1.3.6	citrate-lyase	4

4.2.1.11	phosphopyruvate hydratase	1

4.2.1.76	UDP-glucose 4,6-dehydratase	2

4.4.1.5	lactoylglutathione lyase	3

5.1.3.2	UDP-glucose 4-epimerase	1

5.1.3.3	aldose 1-epimerase	4

5.3.1.5	xylose isomerase	1

5.4.2.1	phosphoglycerate mutase	1

5.4.2.2	phosphoglucomutase	1

5.4.2.8	phosphomannomutase	2

5.5.1.4	inositol-3-phosphate synthase	2

40 E.C. classes were detected among the carbohydrate related unigenes. Each E.C. class is illustrated by a representative member. The number of genes associated to each E.C. class is detailed in the "abundance" column.

**Table 4:** Location of chicory genes related to carbohydrate within metabolic pathways.

Insulin signalling pathway	2.7.1.40	3.1.3.11		
Citrate cycle (TCA cycle)	1.3.99.1	2.3.3.8	4.1.1.49	4.1.3.6

Pentose and glucuronate interconversions	2.7.7.9	3.2.1.15	5.3.1.5	

Inositol metabolism	4.1.2.13			

Alanine and aspartate metabolism	2.4.1.1	2.6.1.1	3.2.1.1	

Purine metabolism	2.7.1.40	5.4.2.1		

Type II diabetes mellitus	2.7.1.40			

Nucleotide sugars metabolism	1.1.1.133	2.7.7.9	4.2.1.76	5.1.3.2

Tyrosine metabolism	2.6.1.1	3.2.1.1		

Two-component system – general	1.3.99.1	4.1.3.6		

Arginine and proline metabolism	2.6.1.1	3.2.1.1		

Phenylalanine metabolism	2.6.1.1	3.2.1.1		

Streptomycin biosynthesis	1.1.1.133	5.4.2.2	5.5.1.4	

Pantothenate and CoA biosynthesis	3.2.1.1			

Urea cycle and metabolism of amino groups	3.2.1.1			

Beta-Alanine metabolism	3.2.1.1			

D-Glutamine and D-glutamate metabolism	3.2.1.1			

Galactose metabolism	2.7.1.6	2.7.7.9	5.1.3.2	5.4.2.2

Heparan sulfate biosynthesis	2.4.1.224	2.4.1.225		

Glycan structures – biosynthesis 1	2.4.1.224	2.4.1.225		

Biosynthesis of siderophore group nonribosomal peptides	3.2.1.1			

Glycolysis/Gluconeogenesis	2.7.1.40	2.7.2.3	3.1.3.11	4.2.1.11

	1.1.1.2	1.2.1.12	2.4.1.1	5.1.3.3

	1.1.1.27	4.1.2.13	5.4.2.2	

Reductive carboxylate cycle (CO_2 _fixation)	1.3.99.1	2.3.3.8		

Butanoate metabolism	1.3.99.1	2.4.1.1		

Carbon fixation	2.6.1.1	2.7.2.3	3.1.3.11	4.1.1.49

	2.2.1.1	2.7.1.40	4.1.2.13	

Glutathione metabolism	1.1.1.49			

Oxidative phosphorylation	1.3.99.1	3.6.3.6		

Caprolactam degradation	1.1.1.2			

Inositol phosphate metabolism	5.5.1.4			

Glycerolipid metabolism	1.1.1.2			

Cysteine metabolism	1.1.1.27	2.6.1.1		

Polyketide sugar unit biosynthesis	1.1.1.133			

Biosynthesis of ansamycins	2.2.1.1			

Valine, leucine and isoleucine biosynthesis	2.4.1.1			

Fructose and mannose metabolism	3.1.3.11	3.1.3.46	5.3.1.5	5.3.1.8

	2.7.7.13	4.1.2.13	5.4.2.8	

Pentose phosphate pathway	1.1.1.49	2.2.1.1	3.1.3.11	4.1.2.13

	5.4.2.2			

Starch and sucrose metabolism	2.4.1.13	2.7.7.7.27	2.7.7.9	3.1.3.12

	3.1.3.24	3.2.1.15	3.2.1.4	5.4.2.2

Pyruvate metabolism	1.1.1.27	2.4.1.1	2.7.1.40	4.1.1.49

	4.4.1.5			

Propanoate metabolism	1.1.1.27			

Glutamate metabolism	2.6.1.1			

Androgen and oestrogen metabolism	1.6.1.1			

Sphingolipid metabolism	1.6.1.1			

Benzoate degradation via CoA ligation	1.3.99.1			

Alkaloid biosynthesis I	2.6.1.1			

Aminosugars metabolism	3.2.1.14			

Novobiocin biosynthesis	2.6.1.1			

Phenylalanine, tyrosine and tryptophan biosynthesis	2.6.1.1			

The 40 E.C. categories related to carbohydrate take part in one or several of the 47 listed metabolic pathways.

Searching the Gene Ontology database [18] with the keyword "carbohydrate" returned 42 GO terms related to molecular functions (8), biological processes (33) or cellular components (1). Each term is associated to a GO number. When searching for these 42 numbers in the 4,608 mapped unigenes, 85 additional carbohydrate related unigenes were retrieved. Among others, these sequences included sugar transporters as well as fructan exohydrolase genes 1-FEH I, 1-FEH IIa and 1-FEH IIb.

### Seasonal evolution of functional classes

Ten chicory root cDNA libraries were produced from tissues sampled during a growing season. Two additional chicory leaf and chicory nodule libraries were also prepared for comparison purpose. For each library, approximately 1,000 clones were randomly chosen and sequenced. While not originating from a clonal population, all these chicory roots originate from a single cultivar (Arancha) and provide an opportunity to compare, *in silico*, the evolution of transcripts in chicory root during the growing season. This implicitly assumes that the abundance of an EST within a dataset reflects the transcription level of the gene in the corresponding organ at the time of sampling, which somehow overlooks the impact of RNA stability on RNA abundance.

To monitor the seasonal evolution of functional classes, the twelve chicory EST datasets passed through a homology and annotation procedure (Blast2GO). Each dataset was treated independently for functional classification. For each dataset, the number of sequences detected in each functional category was recorded giving an estimate of their respective abundance. Because all datasets did not contain the same number of sequences, the abundances were normalized (X/1000) according to the number of Plant GoSlim annotation results in each dataset (table 5).

**Table 5:** BLASTX and GO annotation statistics for the twelve chicory EST datasets

	ESTs	BLASTX ≤ 10^-04^	With GO ID	Blast2GO ann.	Plant GoSlim ann.
FC	994	880 (88%)	863 (87%)	690 (69%)	669 (67%)

NO	968	807 (83%)	781 (81%)	612 (63%)	610 (63%)

RB	967	761 (79%)	740 (77%)	571 (59%)	571 (59%)

RC	1,020	826 (81%)	797 (78%)	622 (61%)	622 (61%)

RE	1,005	895 (89%)	862 (86%)	675 (67%)	675 (67%)

RG	999	672 (67%)	642 (64%)	489 (49%)	488 (49%)

RK	1,013	860 (85%)	827 (82%)	654 (65%)	653 (64%)

RL	988	707 (72%)	678 (69%)	539 (55%)	539 (55%)

RN	1,003	852 (85%)	827 (82%)	641 (64%)	641 (64%)

RO	1,185	794 (67%)	764 (64%)	591 (50%)	590 (50%)

RQ	993	639 (64%)	600 (60%)	458 (46%)	458 (46%)

RS	1,091	878 (80%)	835 (77%)	631 (58%)	631 (58%)

**Av.**	**1019**	**(78%)**	**(75%)**	**(59%)**	**(58%)**

This table provides numerical data concerning the number of sequences remaining after each annotation step.

These analyses were performed for "biological processes" and "molecular function".

Our aim was then to compare the seasonal evolution of biological processes and molecular function to define groups of similarly regulated/expressed functional classes.

Transcriptional clustering analyses were performed with MeV (MultiExperiment Viewer) [19,20]. Functional categories (biological process and molecular function) were clustered with the K-Means method using 1,000 iterations. Hierarchical clustering was performed with Euclidean distances with "average linkage clustering" as linkage method.

**Figure 6:**
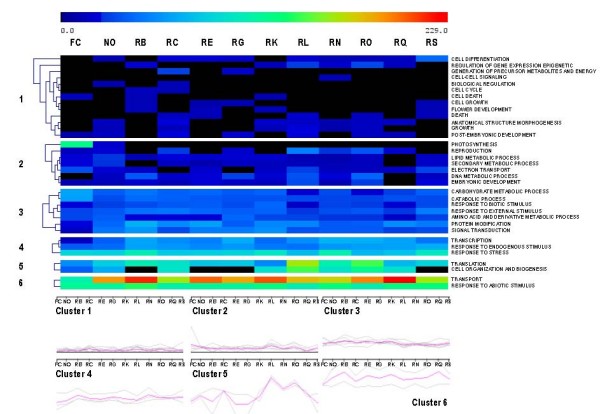
**Seasonal evolution of "biological process" functional classes abundances in chicory roots (Rx), leaves (FC) and nodules (No)**. The color of each square depends on the number of sequences classified in each category. The recorded abundances were comprised between 0 (black) and 229 (red). The second part of the chart represents graphically the same results. All figures were drawn at the same scale.

At the level of biological processes, 34 classes were detected and grouped in six similarly regulated clusters (figure 6).

Since chicory roots are storage structures, the overwhelming abundance of transport related sequences during the growing season could be related to the storage function of the root.

Plants being rooted, the way they respond to biotic and abiotic stimulations is mainly driven by transcriptional regulation since they are unable to adapt their behavior or modify their location. The abundance of sequences in the functional classes related to the response to all types of stimuli (biotic, external, endogenous or abiotic) illustrates this biological adaptation.

**Figure 7:**
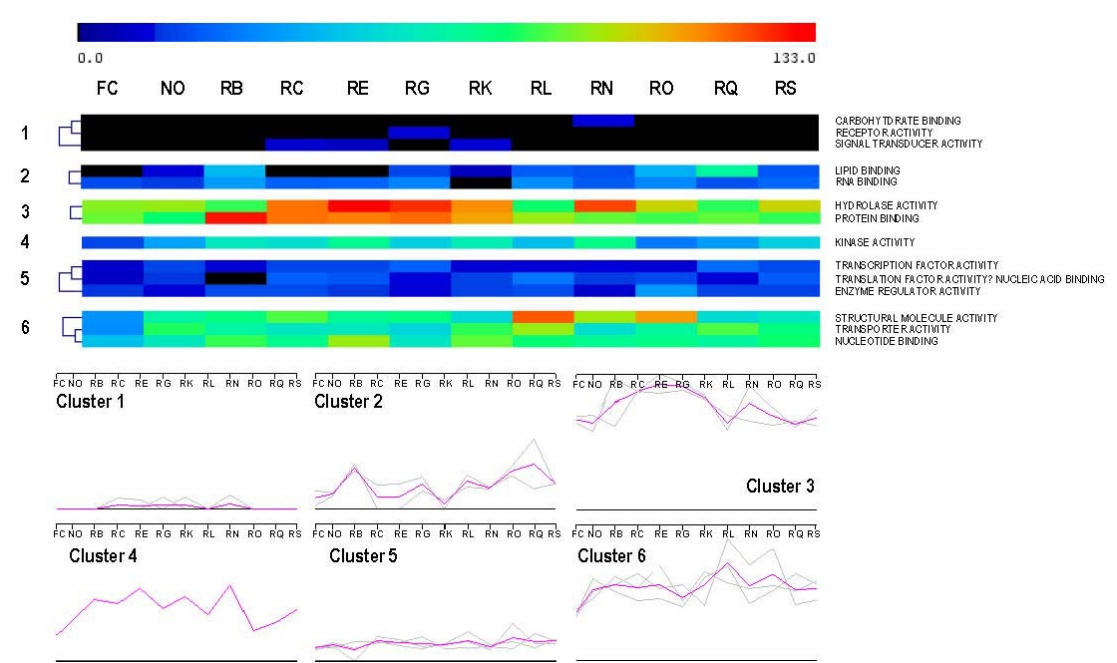
**Seasonal evolution of "molecular function" functional classes abundances in chicory roots (Rx), leaves (FC) and nodules (No)**. The color of each square depends on the number of sequences classified in each category. The recorded abundances were comprised between 0 (black) and 133 (red). The second part of the chart represents graphically the same results. All figures were drawn at the same scale.

At the level of molecular functions, 14 classes were detected and grouped in six transcriptional clusters (figure 7). The most abundant molecular functions refer to "hydrolase activity" and "protein binding" as well as to a second cluster containing "structural molecule activity", "transporter activity" and nucleotide "binding".

### Seasonal evolution of redundant transcripts

Until now, transcriptomic arrays are not available for chicory nor any other type of high throughput transcriptomic tools or results. Despite the relatively small number of ESTs available, we were wondering whether analyzing the distribution and abundance of redundant transcripts during the growing season could give any preliminary insights on the root transcriptome.

This *in silico *Northern analysis aims at detecting, on the basis of sequence data, the evolution of transcript levels.

To test the validity of the *in silico *northern analysis, one of the two key enzymes of inulin synthesis (1-SST) was individually analyzed *in silico*. These results were compared with the seasonal expression pattern studied by northern blot [21]. The 1-SST enzyme is known to be down regulated at the end of the growing season, especially after exposure to cold temperatures, while 1-FFT expression level remains constant. From our *in silico *analysis, 37 sequences of 1-SST were detected in the PHYTOMOL dataset and distributed among the various libraries (table 6).

**Table 6:** *in silico *evolution of the expression of 1-SST during growing season

	Library size	SST	SST/1000
RB	967	1	1.03

RC	1.020	10	9.80

RE	1.005	3	2.99

RG	999	7	7.01

RK	1.013	12	11.85

RL	988	0	0,00

RN	1.003	2	1.99

RO	1.185	2	1.69

RQ	993	0	0,00

RS	1.091	0	0,00

The table describes the total number of sequence in each dataset, the number of 1-SST sequences that were detected and their normalized abundance (x/1000).

From these results, a clear fall in the abundance of 1-SST transcripts was observed starting from the RL library (fall from 12 to 0 sequences). This fall corresponds to the time when the plant was exposed to minimal temperatures close to 0°C (around sampling RK). The second drop corresponds to the exposure to sub-zero temperatures (just before sampling RQ).

These results are in complete agreement with previously published northern blots [21] as well as with semi-quantitative results (unpublished). Differences between time points can probably be explained by the fact that the roots used to produce the libraries originated from a variety and not from a clonal population, something already observed on northern blot results [21].

No other information concerning the seasonal evolution of chicory genes expressed in the root is presently available, or genes for which the information is available are not sufficiently represented in the libraries to allow *in silico *northern analysis (in particular, 1-FFT and the FEH multigene family).

From the twelve datasets, all ESTs were compared to generate groups of identical/homologous sequences (sequence clustering): a total of 465 clusters were identified, each containing at least 4 (arbitrary defined) and up to 90 sequences.

For each cluster, sequences were further subdivided according to their library of origin. Abundance was normalized depending on the number of clones sequenced from each library. Detailed results obtained for the 465 gene clusters are not presented but are available upon request.

Table 7 presents the top10 largest EST clusters. Homology searches performed with all available BLAST analyses (sequence based homology search) and Pfam (protein conserved domains based homology search) in order to identify the most abundant cluster (90 sequences) gave, until now, no significant results.

**Table 7:** List of top 10 largest EST clusters

Cluster size	BLASTX results
90	No hit

75	Auxin-regulated protein [*Arabidopsis thaliana*]

74	Catalase3 [*Helianthus annus*]

63	Ribulose-1,5-bisphosphate carboxylase [*Lactuca sativa*]

57	Dehydrin [*Phaseolus vulgaris*]

48	P-rich protein eig-i30 [*Nicotiana tabacum*]

41	Vegetative storage protein VSP [*Cichorium intybus*]

39	Asparaginyl endopeptidase [*Vigna mungo*]

39	Heat shock protein 70 [*Saussurea medusa*]

38	Sucrose: sucrose 1-fructosyl transferase [*Cichorium intybus*]

The sequence clustering results highlight 31 gene clusters which only appear in the leaf and nodule libraries. The nodule library was obtained after tissue culture of chicory witloof leaves. As expected, these sequences were mainly associated to photosynthesis. No nodule specific clusters were identified.

**Figure 8:**
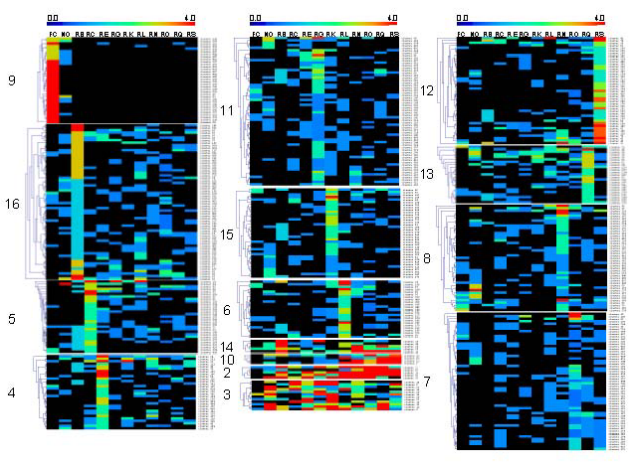
***In silico *northern analysis of the seasonal evolution of abundant gene clusters in chicory roots (Rx), leaves (FC) and nodules (No)**. The color of each square depends on the frequency of each gene in each library. The values were comprised between 0 (black) and 4 (red). The second part of the chart represents graphically the same results. All figures were drawn at the same scale.

Using the MeV software, the 465 sequence clusters (each sequence cluster represents a single unigene isolated several times, out of a single or multiple cDNA libraries) were grouped in 16 expression clusters. One expression cluster groups sequences with similar expression pattern. Since cluster 1 only contained a single sequence, this cluster was discarded. The remaining 15 clusters are shown in figure 8.

The two first columns correspond to the leaf and nodule EST databases, while the following ten columns correspond to the ten root databases positioned according to their sampling date (ascending).

Cluster 9 contains sequences nearly exclusively expressed in chicory leaf and nodule. These sequences appear to be linked to photosynthesis.

Among clusters containing sequences exhibiting strong expression in one or several contiguous datasets, one can distinguish clusters 2 and 10. They both contain sequences highly expressed at the end of the growing season. Cluster 10 contains sequences presenting amino-acid sequence homology to a putative dehydrine [GenBank: CAC80717], two chicory vegetative storage proteins [GenBank: CAB71301] and one sequence with homology to pectin-methylesterase [GenBank: NP_201041].

Cluster 2 contains sequences with homology to a dehydrine [GenBank: T11744], a catalase from Helianthus annuus [GenBank: AAF61733] and the most abundant sequence of the entire dataset (90 occurrences) which remains until now unidentified.

These results indicate preferential expression of sequences involved in response to cold stress (dehydrine) and detoxification of reactive oxygen species (catalase) at the end of the growing season. Storage proteins are also abundant since three chicory vegetative storage proteins were detected.

Cluster 12 contains several sequences significantly overexpressed in the RS dataset obtained from samples harvested late in the growing season, after exposure to cold temperatures. These sequences present homology to a dehydrin [GenBank: NP_177745], an ABA-responsive protein [GenBank: NP_568744], a mitogen activated protein kinase 3 [GenBank: AAP20421], a spliceosome associated protein [GenBank: NP_193897], a cysteine protease [GenBank: BAD10859], an RNA binding protein homolog [GenBank: T01932], a ubiquitin-associated uba/pb1 domain containing protein [GenBank: NP_194200], a quinone oxydoreductase homolog [GenBank: T11672], a hexameric polyubiquitin [GenBank: AAA34123], an elongation factor beta-1 [GenBank: NP_179402] and a protein WSI724 water stress induced [GenBank: T07613]. These sequences highlight the presence of proteases and protein degradation through ubiquitination. Water and cold stress associated proteins are also detected such as dehydrin, a water stress induced and an ABA-responsive protein.

The biological meaning of most of the other clusters remains however still speculative, since they were defined on the basis of a few sequences out of each library. In addition, the clustering process can misleadingly associate together two closely related but distinct sequences. In consequence, these results have to be taken as indicative and still need to be further investigated.

## Discussion

Expressed Sequence Tags (EST) are tools of choice to investigate the expressed portion of an uncharacterized genome. Prior to this publication, only two datasets were publicly released: 3,348 ESTs from chicory somatic embryo (landrace Koospol) published in 2007 [22] and 38,356 ESTs from the CGPDB released on February 2007 (Ames 3210 #2 from the Netherlands, R. Kesseli, personal communication) [23]. These two datasets have now been merged into a single database of 41.704 ESTs, representing, according to their analysis, 22,038 putative unigenes. The PHYTOMOL dataset represents the second most important EST collection for chicory and is unique in that it consists of 12 distinct libraries of genes mainly expressed in the roots during one entire growing season. The non redundant PHYTOMOL dataset consists of 1,922 contigs and 4,869 singlets for a total of 6,791 unigenes according to EGassembler analysis results. To evaluate the level of redundancy between the PHYTOMOL and the CGPDB datasets, all the sequences (53,930) passed through the EGassembler clustering pipeline: a total of 23,135 unigenes were retrieved, which is slightly more than the number of unigenes present on the CGPDB website (22,038). Performing the clustering of the 41.704 chicory ESTs from the CGPDB dataset with EGassembler returns 19,621 unigenes (7,893 contigs and 11,728 singlets). This indicates that the parameters used by CAP3 in the EGassembler pipeline may underestimate the effective diversity of the dataset. As observed from the result list, for these last tests, and under CAP3 clustering conditions used by EGassembler, our chicory dataset contains 3,512 unigenes which were present neither in the somatic embryo EST database, nor in the original CGPDB chicory dataset. We believe that these additional unigenes do not result from low quality sequences but rather from extensive sampling of genes expressed in the root throughout the growing season. This assumption of non exhaustivity of the CGPDB was also reported by Legrand [22], who mentions that 31% of the chicory ESTs expressed in somatic embryos did not yield any significant homology results when compared to the Asteraceae databases.

Concerning the SNP discovery rate in the PHYTOMOL dataset, it varies depending on the SNP selection criterions used by the CGPDB detection pipeline and during our manual editing. Since we only investigated the PHYTOMOL dataset, it is likely that including other available chicory ESTs would increase the number of putative SNPs that could be retrieved by datamining.

Among the 6,791 putative unigenes of the PHYTOMOL dataset, 4,811 (70.8%) returned a significant BLASTX result, which is 16% less than the 87% reported by Legrand for chicory somatic embryo ESTs [22]. The functional annotation of these unigenes highlighted "transport" as the most diversified class of biological processes. In chicory, most photosynthates have to be exported through the phloem and unloaded into the root, which is probably why transporter and protein binding activities were diversified and abundant in chicory roots.

Many different sequences were also associated with "hydrolase activity" as well as "protein binding" early in the season (RC to RK samplings), at a period when chicory roots only started to accumulate carbohydrates. This period corresponds to a time of high energy demand, which could require massive sugar degradation to cope with metabolic needs.

Most other classes of molecular functions were evenly abundant during the growing season. This apparent transcript level stability reflects abundance steadiness rather than sequence identity within a functional category: the nature of the sequences could change within a functional category while the average abundance of this category remained constant along the season.

Despite this limitation, the PHYTOMOL dataset was also used for *in silico *Northern analyses of 465 individual sequences, classifying them into 15 clusters of presumably similarly regulated genes (figure 8). Clusters 2, 10, 12 and 13 contain sequences expressed after exposure to cold temperature. In these four clusters, a clear overexpression of cold and water-stress related sequences (dehydrine and water stress-induced proteins) was observed as expected. A good correspondence was also found between *in silico *analysis of 1-SST abundance in the PHYTOMOL dataset and literature reports. However, these observations have to be considered as preliminary and indicative since most unigenes contained only a limited number of sequences which limits the power of this analysis.

One of the aims of this study was to isolate genes related to carbohydrate metabolism. The EST approach was indeed successful in isolating more than 200 chicory partial sequences of such genes expressed mainly in the root.

## Conclusion

Expressed sequence tags (EST) are valuable tools for genomic research in non model species such as industrial chicory. This project allowed generation and characterization of more than 12,000 ESTs obtained from twelve chicory libraries, ten of which from roots sampled during the growing season. The resulting dataset yielded nearly 6,800 putative unigenes, from which over 70% were presenting BLASTX homologies to publicly available sequence resources.

Recently, two other EST datasets were released, pinpointing the increasing interest of the scientific community for Asteraceae as proven by the set-up of the Compositae Genome Project Database. Our contribution yielded more than 3,500 new unigenes.

When analyzing the sequence clusters produced to generate the contigs, abundance of polymorphisms were detected: simple sequence repeats, insertions, deletions as well as Single Nucleotide Polymorphisms. The use of this genic polymorphism for molecular marker design will be considered in the future for breeding purpose.

## Methods

### Plant Material

Industrial chicory (cultivar Arancha), was grown in a field in Grand-Manil (Gembloux, Belgium). Weeds were mechanically eliminated. Roots and leaves were sampled between August 21, 2002 and February 07, 2003 (figure 1). Once uprooted, roots were carefully washed, pealed and cut in 10 g blocks which were immediately immerged in liquid nitrogen and kept at -80°C until further use. Leaves were collected on September 03, 2002 and immediately frozen in liquid nitrogen. Nodules were produced from the selected micropropagated clone '4/11' of C. intybus c.v. "witloof". Nodulation was induced on wounded leaves cultivated in vitro as described by Piéron et al. [24]. Nodules were sampled 45 days after induction and immediately frozen in liquid nitrogen. All samples were stored at -80°C.

### cDNA library construction

Total RNA was extracted by TRIzol^® ^Reagent (GibcoBRL) from chicory roots or leaves ground in liquid nitrogen with mortar and pestle. Poly(A)+ messenger RNAs were further purified on an oligodT cellulose column according to manufacturer's instructions (Amersham Pharmacia Biotech). The cDNAs were synthesized, size fractionated and directionally cloned into the EcoRI/XhoI sites of Uni-Zap^®^XR vector using a ZAP-cDNA^® ^Synthesis kit and a ZAP-cDNA Gigapack^® ^III Gold Cloning Kit (Stratagene). Libraries were characterized and stored at both 4°C and -80°C in 7% DMSO (V/V). cDNA libraries were designated by two letters: the first letter refers to the organ of origin: leaf (F), root (R) or nodule (N) and the second letter referred to the sampling date, B to S (figure 1).

Ten different root cDNA libraries, one leaf cDNA library and one nodule cDNA library were generated. Figure 1 presents the evolution of the temperatures and the ten selected field sampling dates. Sampling dates "O" and "Q" are respectively positioned just before and after the first exposure to sub-zero temperatures.

### Plasmid excision

The cDNAs were excised in the pBluescriptSK phagemid using the ExAssist helper phage with the bacterial strain SOLR (Stratagene). Bacterial clones were obtained by infection of SOLR bacterial strain with pBluescript containing phagemids. Plasmid preparations were used as template to sequence the cDNA inserts from the 3'end.

### Bacterial culture conditions and plasmid DNA isolation

Sequencing was performed on plasmid DNA. Overnight bacterial cultures were started from freshly grown isolated bacterial clones, at 37°C, in 2.2 ml 96 wells plates (Westburg AB-0661) with 1.2 mL "Circle grown" culture medium (P's Serving Life Sciences 3000-112). The culture plates were sealed with a gas permeable adhesive foil (Westburg AB-0626) and strongly agitated (290 rpm). A glycerol stock of each clone was stored at -80°C. From the rest of the culture, plasmids were purified using a typical alkaline lysis procedure and filtration plates (Millipore Multriscreen Filtration System MAGVN2250) to remove protein and cellular debris prior to the plasmid precipitation step.

### Sequencing

Sequencing was mainly performed from the 3' end of the inserts, starting from 300 ng plasmidic DNA; some 5' sequencing was also performed for sequences belonging to abundant unigene clusters. Primer RvM13pbs 5'-GGA-AAC-AGC-TAT-GAC-CAT-GA-3' was used for 5'end sequencing and FwM13pbs 5'-GAT-AAA-CGA-CGG-CCA-GTG-3' for 3'end sequencing. Sequencing reactions were analyzed on a ABI 3100 Genetic Analyzer (Applied Biosystems, Foster City, USA), a CEQ8000 Genetic Analyzer (Beckman Coulter, San Diego, USA) or a Li-Cor global edition IR2 DNA sequencer (Westburg).

Sequencing reactions were performed, respectively using the "Big Dye Terminator" kit, version 1.1 (Applied Biosystems), Genome Lab DTCS Quick start kit (Beckman Coulter) and DYEnamic Direct Cycle Sequencing kit (GE Healthcare).

### Generation of the unigene dataset

The generation of a unigene dataset produces a set of non redundant sequences composed of singlets (sequences present as a single copy in the dataset) and contigs (consensus sequences obtained after alignment of a set of homologous or identical sequences). Generating a unigene dataset requires several steps, all of them integrated in a freely accessible web-interface named "EGassembler" [25]. For the unigene dataset generation, EGassembler was used with default analysis parameters. This bio-informatic pipeline starts with a sequence cleaning stage, removing low quality sequence stretches, followed by the detection and removal of repetitive elements.

The resulting output is then searched and cleaned from organelle sequences. The remaining sequences are compared to generate homology clusters and deduce contig sequences thanks to the CAP3 software [26]. The sequences not included in clusters were classified in the singlets dataset.

### SNP datamining in the PHYTOMOL dataset

SNP polymorphism was performed using the procedure and the scripts available on the CPGDB [16].

### Homology searches and annotations

All homology and annotation searches were performed with Blast2GO [17,27]. The BLASTX cut off value was set to 10^-5^. Mapping was the second step of the annotation pipeline. For the mapping process, previously validated settings were used: Evalue 10^-5^, annotation Cut Off "45" and GO Weight "10" (for more details on the validation process, refer to the Blast2GO website). These settings give the best balance between annotation rate and accuracy. The annotation step retrieves keywords in the BLASTX results description and converts them into Gene ontology (GO) related terms. The last step called "annotation" tends to define a consensus GO classification from all the retrieved GO related terms. This annotation was simplified and focused on plant related functional categories by using the Plant GOslim annotation conversion option of the Blast2GO software.

### *In silico *expression profiles

*In silico *expression profile clusterings were performed with MultiExperiment Viewer [19,20].

## Authors' contributions

DM performed the samplings and the construction of the libraries. Sequencing was performed by BP, ND and DM. ND performed the annotation and data mining as well as most of the preparation of the manuscript. CM performed the bio-informatic investigation of SNP polymorphism. MB, BW and PVC conceived and supervised these studies and data analyses. All authors read and approved the final manuscript.
